# Are geometric morphometric analyses replicable? Evaluating landmark measurement error and its impact on extant and fossil *Microtus* classification

**DOI:** 10.1002/ece3.6063

**Published:** 2020-03-13

**Authors:** Nathaniel S. Fox, Joseph J. Veneracion, Jessica L. Blois

**Affiliations:** ^1^ Environmental Systems Graduate Group University of California Merced CA USA; ^2^ Department of Life and Environmental Sciences University of California Merced CA USA

**Keywords:** discriminant analysis, error, geometric morphometrics, landmarks, *Microtus*

## Abstract

Geometric morphometric analyses are frequently employed to quantify biological shape and shape variation. Despite the popularity of this technique, quantification of measurement error in geometric morphometric datasets and its impact on statistical results is seldom assessed in the literature. Here, we evaluate error on 2D landmark coordinate configurations of the lower first molar of five North American *Microtus* (vole) species. We acquired data from the same specimens several times to quantify error from four data acquisition sources: specimen presentation, imaging devices, interobserver variation, and intraobserver variation. We then evaluated the impact of those errors on linear discriminant analysis‐based classifications of the five species using recent specimens of known species affinity and fossil specimens of unknown species affinity. Results indicate that data acquisition error can be substantial, sometimes explaining >30% of the total variation among datasets. Comparisons of datasets digitized by different individuals exhibit the greatest discrepancies in landmark precision, and comparison of datasets photographed from different presentation angles yields the greatest discrepancies in species classification results. All error sources impact statistical classification to some extent. For example, no two landmark dataset replicates exhibit the same predicted group memberships of recent or fossil specimens. Our findings emphasize the need to mitigate error as much as possible during geometric morphometric data collection. Though the impact of measurement error on statistical fidelity is likely analysis‐specific, we recommend that all geometric morphometric studies standardize specimen imaging equipment, specimen presentations (if analyses are 2D), and landmark digitizers to reduce error and subsequent analytical misinterpretations.

## Graphical abstract

We replicated the same specimen dataset multiple times to evaluate landmark data acquisition error on the lower first molar of five North American vole species. Results indicate that geometric morphometric error can be substantial within and among data acquisition trials. Our study emphasizes the need to standardize specimen presentations (if analyses are 2D) and landmark digitizers when conducting geometric morphometric studies to reduce error and subsequent analytical misinterpretations.

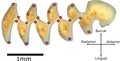

## 1 INTRODUCTION

Geometric morphometrics (GM) is a popular technique for evaluating shape and shape change among biological specimens. It is often used in ecology, archeology, and paleontology to address a variety of topics including taxonomy (De Meulemeester, Michez, Aytekin, & Danforth, 2012; Jansky, Schubert, & Wallace, 2016; Wallace, 2006), ecomorphology (Cassini, 2013; Curran, 2012; Figueirido, Palmqvist, & Pérez‐Claros, 2009; Gómez Cano, Hernández Fernández, & Álvarez‐Sierra, 2013; Meachen, Janowicz, Avery, & Sadleir, 2014), evolution and development (Lawing & Polly, 2010), and population history (Baumgartner & Hoffman, 2019; Bignon, Baylac, Vigne, & Eisenmann, 2005; Gaubert, Taylor, Fernandes, Bruford, & Veron, 2005; Nicholson & Harvati, 2006). Geometric morphometric “shape” is quantified via Cartesian landmark coordinate configurations positioned on discreet, biological loci (Zelditch, Swiderski, Sheets, & Fink, 2004). The scale, location, and rotational orientation of these landmark configurations are then standardized via generalized Procrustes analysis (GPA) superimposition to isolate and compare object shape (Kendall, 1977; Rohlf & Slice, 1990). Geometric morphometric analysis of specimens projected on 2D and 3D surfaces can be advantageous over qualitative morphological descriptions and traditional morphometrics (e.g., linear measurements) since the former is often subjective and the latter is correlated with object size (Schmieder, Benítez, Borissov, & Fruciano, 2015). Unlike traditional morphometrics, GM also excels at shape visualization which facilitates communication of empirical results (Zelditch et al., 2004).

Despite its analytical advantages and broad utility, replicating GM results can be challenging due to the variety of research equipment used to image the samples, variation in specimen positioning, and variation in landmark digitization within and among operators, all of which can generate data discrepancies. Each phase of GM data acquisition can introduce a unique form of random and/or systematic measurement error. When compounded, these errors may lead to inconsistency among repeated measures and obscure the distinction between biological and artificial variation among specimens (Fruciano, 2016; Robinson & Terhune, 2017). Three general types of GM‐based measurement error are acknowledged (methodological, instrumental, and personal), which can be subdivided into more specific error sources (Arnqvist & Mårtensson, 1998). Here, we address four sources of measurement error encountered during landmark data acquisition:

### 1.1 Imaging device; error type: Instrumental

Use of different instruments for projecting 3D objects on 2D and 3D surfaces (e.g., digitizing tablets, digital images, and scanners) can generate dissimilar morphological reconstructions of original specimens (Arnqvist & Mårtensson, 1998). Variation can occur within equipment types as well. Camera lenses, for example, generate 2D image distortion based on the magnification of an object and its distance and position from the camera; the extent of image distortion varies among lens types due to factors such as lens curvature (Zelditch et al., 2004). The resolution of an image will also vary depending on the number of photodetectors in a camera (Zelditch et al., 2004); some anatomical loci may be obscured in lower‐resolution images which can impact the precision of landmark placement. Error facilitated by dimensional loss is specific to 2D GM analyses; however, other forms of instrumental error can occur in 3D systems as well (Fruciano et al., 2017; Robinson & Terhune, 2017). The configuration of landmarks placed on specimen images may, therefore, be inconsistent when different equipment is used and/or when data from different imaging protocols are combined.

### 1.2 Specimen presentation; error type: Methodological

Operators digitizing specimens in two dimensions should be cautious of their presentations (i.e., the projected orientation of specimens) since some degree of distortion is usually unavoidable when projecting 3D objects. Differential shifting of three‐dimensional features can be problematic in 2D systems because *z*‐axes are not retained and, therefore, projected locations of landmark loci can be displaced relative to their true position among other loci (Buser, Sidlauskas, & Summers, 2018; Cardini, 2014; Zelditch et al., 2004). Effects of such displacement can be exacerbated if landmark loci shift toward the edges of a camera field where image distortion is greatest (Fruciano, 2016; Zelditch et al., 2004). If all objects are projected from similar orientations and with the same equipment, any projection distortions should be similar among specimens and are thus unlikely to generate substantial artificial variation. If presentations are dissimilar among species, however, associated interspecimen variation in landmark coordinates may appear biological in downstream analysis when it is in fact artificial. Presentation error may be particularly substantial in situations where interspecimen orientations are difficult to standardize (e.g., when comparing within‐cranium teeth of recent specimens to isolated teeth of fossil specimens).

### 1.3 Interobserver error; error type: Personal

After specimens have been selected, presented, and projected, error can occur during landmark digitization. For example, one individual may position a landmark differently than another individual, even when digitizing the same locus of the same specimen. Error among landmark digitizers is referred to as interobserver error.

### 1.4 Intraobserver error; error type: Personal

Digitizing error occurs within observers as well. An individual may place a landmark on a locus differently from one specimen to another or from one digitizing session to another. This is referred to as intraobserver error. Intra‐ and interobserver error can be affected by factors such as variation in digitizing experience among observers, the number of digitizing sessions conducted per observer, and ease of landmark loci visualization (Fruciano, 2016; Osis, Hettinga, Macdonald, & Ferber, 2015). Observer errors may be exacerbated by variation in specimen projection and/or presentation as well.

Measurement error introduced at various phases of landmark data acquisition can be substantial; however, their collective impacts on GM analyses are underreported (Fruciano, 2016). It is not uncommon for studies to report inter‐ and intraobserver error (e.g., Dujardin, Kaba, & Henry, 2010; Gonzalez, Bernal, & Perez, 2011; Nicholson & Harvati, 2006; Ross & Williams, 2008), possibly because landmark digitization does not require presentation and projection replications and because it can be conducted any time after projected specimen data collection. Personal digitizing errors are therefore more convenient to quantify than most other error types (Fruciano, 2016). Quantification of presentation and projection error requires replication which generally must be conducted at specimen housing facilities, and is seldom assessed in the literature (but see Fruciano, 2016; Fruciano et al., 2017; Robinson & Terhune, 2017) though their potential for obscuring biologically meaningful shape variation is considerable (Fruciano, 2016; Zelditch et al., 2004). Few studies demonstrate how several of these error types can combine to impact statistical result‐based inferences (but see Fruciano et al., 2017,2020; Robinson & Terhune, 2017; Vergara‐Solana, García‐Rodríguez, & Cruz‐Agüero, 2014). This context is important because ecological, archeological, and paleontological studies often use statistical grouping analyses (e.g., linear discriminant analysis (LDA)/canonical variate analysis) to determine the taxonomic or ecological affinity of unknown specimens (Kovarovic, Aiello, Cardini, & Lockwood, 2011; Webster & Sheets, 2010). Despite the frequency of GM‐based classification analyses in the literature (e.g., Baumgartner & Hoffman, 2019; Cassini, 2013; Curran, 2012; De Meulemeester et al., 2012; Gómez Cano et al., 2013; Wallace, 2006), the impacts of multiple sources of measurement error on statistically derived group membership predictions are largely untested.

Here, we evaluate the relative contribution of GM measurement error from different landmark data acquisition sources and their impact on LDA group membership predictions. We specifically quantify error introduced from four sources—specimen presentation, specimen imaging devices, interobserver digitization, and intraobserver digitization—and determine how the accuracy and replicability of 2D landmark‐based identifications of five closely related extant species, and the predicted group membership replicability of congeneric specimens of unknown species affinity, are affected by each error source. For this study, we define “replicable” as achieving the same group membership predictions of individual specimens among repeated data acquisition iterations. We do this so future researchers classifying specimens via landmark analysis are aware of (a) the data acquisition sources that may introduce non‐negligible amounts of measurement error and (b) the precautions that can be employed to mitigate those errors and their impacts on statistical results.

## 2 MATERIALS AND METHODS

### 2.1 Study system

Recent work on observer and method‐based GM error suggests that error may have a substantial impact on statistical results when variation among similar (e.g., intraspecific) groups is analyzed because morphological differences among those groups are likely to be subtle (Robinson & Terhune, 2017). Thus, artificial variation introduced via GM error may be more likely to impact classification statistics, and bias subsequent inferences of biological variation, when among‐group variation is low (Robinson & Terhune, 2017). Comprehensive analysis of different types of measurement error and their impact on closely related/morphologically similar group differentiation is seldomly conducted. To explore this, we examine five *Microtus* (vole) species (*Microtus californicus*, *Microtus longicaudus*, *Microtus montanus*, *Microtus oregoni*, and *Microtus townsendii*) distributed throughout western North America. Voles are frequently used as biochronologic and paleoenvironmental indicators at fossil sites due to their habitat specificity and ubiquity in modern and prehistoric biotic assemblages (Bell & Bever, 2006; Bell & Repenning, 1999; McGuire, 2011; Smartt, 1977; Wallace, 2019). However, identifying voles to species is challenging due to high morphological variability, high diversity, and sympatry of species throughout much of North America (Barnosky, 1990; Bell & Bever, 2006; Smartt, 1977; Wallace, 2006). Over the past two decades, more advanced research techniques including landmark‐based LDA of *Microtus* lower first molars (m1s) have improved vole species identification accuracy (Wallace, 2006), but it is still imperfect when applied to study regions such as western North America due to marked geographic range and shape overlap among the many members that reside there today (McGuire, 2011). Western North American voles are therefore an appropriate system for evaluating GM measurement error on classification statistics when intergroup variation is low.

### 2.2 Study design

**Figure 1:**
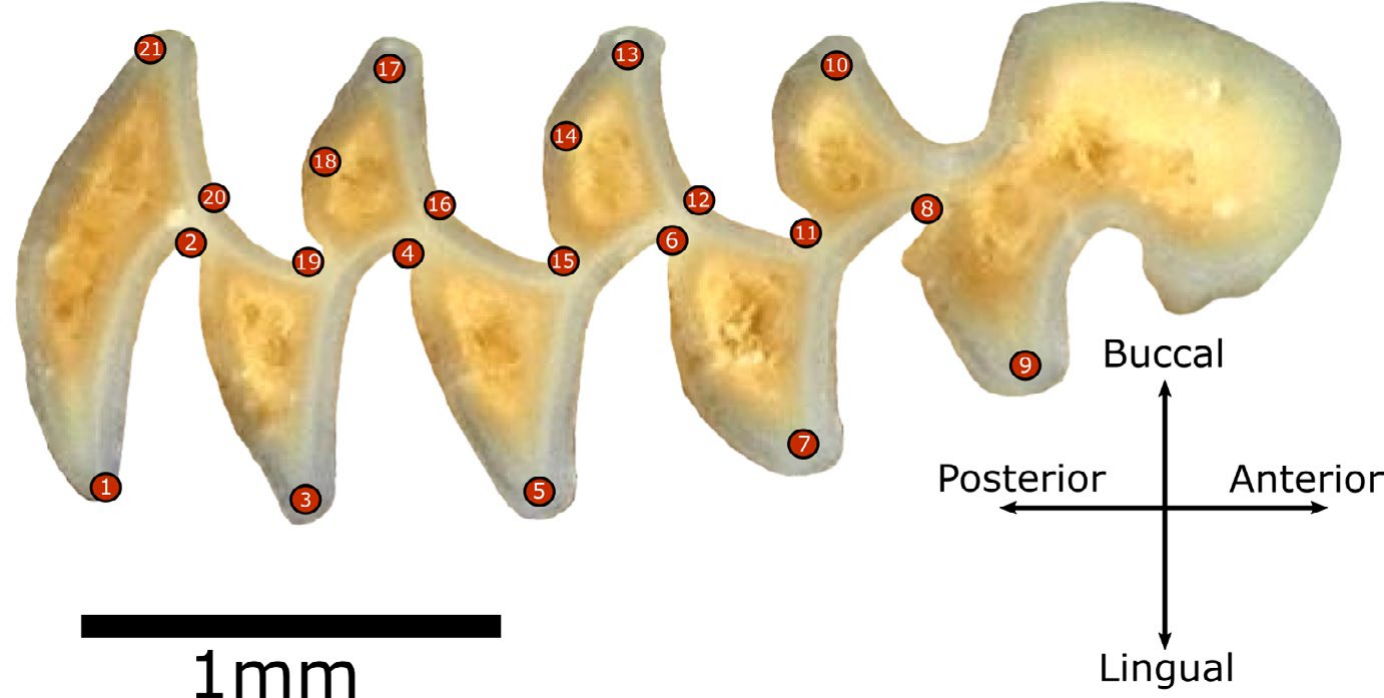
Lower left first molar occlusal surface of MVZ‐132727 *Microtus californicus* illustrating the 21‐landmark configuration used to quantify shape variation among extant and fossil *Microtus* species. See Wallace (2006) for landmark definitions

We replicated 2D digital specimen images (*n* = 247) and m1 landmark configurations (*n* = 21 landmarks, Figure 1) of McGuire (2011) to quantify measurement error from four data acquisition sources and its impact on *Microtus* species classification. All photographed specimens are from the University of California Museum of Vertebrate Zoology (MVZ); see Appendix I of McGuire (2011) for a list of the recent *Microtus* specimens included. We were unable to acquire four of the 251 original specimens from McGuire (2011) (MVZ: 68521, 83519, 96735, 99283) so the final number of individuals analyzed per species is as follows: *M. californicus* (*n* = 49), *M. longicaudus* (*n* = 49), *M. montanus* (*n* = 48), *M. oregoni* (*n* = 50), and *M. townsendii* (*n* = 51). Each species group thus meets ideal LDA conditions that (a) predictor variables (i.e., *x* and *y* Cartesian landmark coordinates, *n* = 42) do not exceed *n* of the smallest group and (b) that group samples sizes are approximately equal (Kovarovic et al., 2011). Each phase of landmark data acquisition (i.e., specimen presentation, specimen imaging, and inter/intraobserver digitization) was repeated to quantify error from those sources. Our study design for quantifying error from each source was as follows:

#### 2.2.1 Imaging device

We assembled two datasets using specimen images obtained from two different cameras to evaluate interinstrument variation (hereafter “imaging device” or simply “device” variation). The first image set included the original *Microtus* dentary images photographed with a Nikon D70s (hereafter Nikon) from McGuire (2011). The second image set included the same specimens photographed with a Dino‐Lite Edge AM4815ZTL Digital Microscope (hereafter Dino‐Lite). Efforts were made to replicate the original Nikon specimen orientations, especially projected angles of occlusal tooth surfaces and specimen distances from the camera lens, to minimize presentation error during this iteration. However, presentation error is necessarily a residual component of imaging device error in 2D systems.

#### 2.2.2 Specimen presentation

After an initial Dino‐Lite photograph was taken, each *Microtus* specimen was tilted haphazardly along its anteroposterior and/or labiolingual axis and rephotographed with all landmark loci still visible. This was done to simulate specimen orientation changes that may occur when comparing dissimilar specimens such as in situ teeth and isolated teeth. That scenario is not uncommon when comparing fossil specimens to recent specimens since complete preservation of fossilized craniodental remains is rare. When loose m1s were available from recent *Microtus* specimens, those teeth were photographed in isolation rather than in situ during this iteration. We note, however, that intentionally tilting specimens potentially exacerbates presentation error relative to the amount of error typically introduced when specimen orientations are standardized. The intent of this modification is to quantify potential presentation error rather than expected error since presentation error will vary by study (Fruciano, 2016).

#### 2.2.3 Inter/intraobserver error

To quantify observer variation, the original Nikon *Microtus* m1 images and Dino‐Lite resampled images were digitized by two observers using the 21‐landmark protocol of Wallace (2006) and McGuire (2011) (Figure 1). Those observers also allowed us to evaluate methodological experience, a variable suggested (but rarely tested) to impact the magnitude of observer error (Fruciano, 2016). It is perhaps expected that experience will reduce digitizing error, but recent studies have shown that is not always true (e.g., Engelkes et al., 2019), thus warranting its quantification here. One observer, hereafter referred to as the experienced observer (EO), had previous experience conducting 2D landmark analyses at the time this study was initiated while the other observer, hereafter referred to as the new observer (NO), did not. Each image set was then digitized a second time by the EO and NO with at least 1 week between iterations to evaluate intraobserver variation on landmark placement.

### 2.3 Data preparation

**Figure A1:**
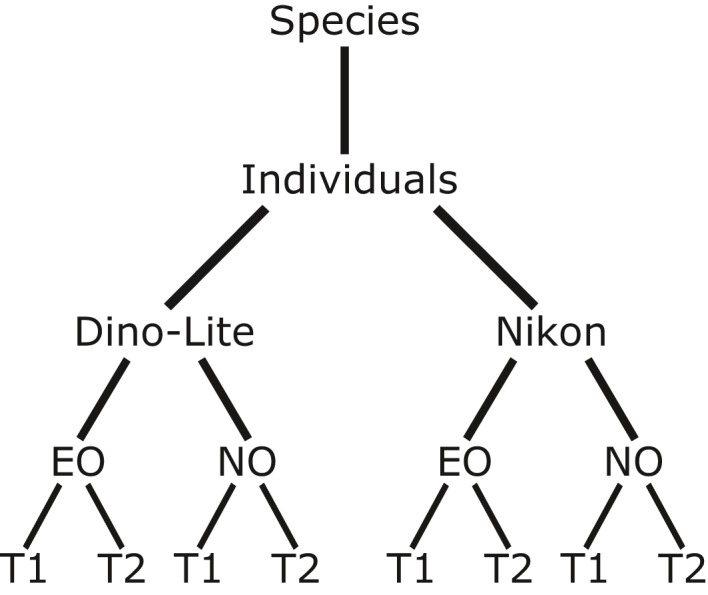
Hierarchical structure for our nested Procrustes ANOVA with results depicted in Table 2

Nine unique landmark datasets were assembled in total to evaluate measurement error from the four focal data acquisition sources. First, Nikon and Dino‐Lite image sets were assembled to quantify imaging device variation. Those image sets were digitized twice by each observer to evaluate inter‐ and intraobserver error (two image sets and two digitizing iterations per observer = eight datasets, Figure A1). A “tilted” Dino‐Lite image set was then assembled and digitized by the EO to quantify data variation due to changes in specimen presentation resulting in a total of nine datasets. All image sets were assembled and digitized using TpsUtil 32 (Rohlf, 2018a) and TpsDig 2.32 (Rohlf, 2018b) software, respectively. Each landmark dataset was superimposed via GPA to standardize effects of rotation, orientation, and scale among specimens using the gpagen function in the R package “geomorph” (version 3.1.3, Adams, Collyer, & Kaliontzopoulou, 2019). During GPA, all specimens are translated to the origin, scaled to unit‐centroid size, and optimally rotated via a generalized least‐squares algorithm to align them along a common coordinate system (Rohlf & Slice, 1990).

### 2.4 Quantifying measurement error

We ran Procrustes ANOVA models using the procD.lm function in geomorph to analyze source‐specific variation in the nine GPA‐transformed landmark datasets. Analyses were conducted on 22 unique pairwise dataset comparisons (see dataset comparison names in Table 1 for specific comparisons) and cumulatively across eight datasets using the following nested hierarchal levels: species > individuals >imaging device > interobservers > intraobservers (Figure A1). Specimen presentation was only evaluated via pairwise comparison of tilted versus nontilted Dino‐Lite datasets because tilted presentations were not included in the original Nikon‐image study design of McGuire (2011). All Procrustes ANOVAs were conducted using a residual randomization procedure with 999 iterations. Dataset comparisons were grouped according to the specific data acquisition iterations they encompassed using a four‐part naming system. For example, “Dinolite_NoTilt_EO_T1” indicates that (a) the images were photographed with a Dino‐Lite camera, (b) the images were not randomly tilted, (c) images were digitized by the EO, and (d) it was the EO's first digitizing iteration. Thus, pairwise comparison of datasets “Dinolite_Notilt_EO_T1” and “Dinolite_Notilt_EO_T2” quantifies intraobserver variation between digitizing iterations one and two for the EO since all other source components are equivalent. In addition, we calculated repeatability among our datasets using pairwise Procrustes ANOVA mean squares based on the protocol and equations of Arnqvist and Mårtensson (1998) and Fruciano (2016). Repeatability quantifies the variability of repeated measurements within the same samples, in this case the resampled *Microtus* datasets, relative to the variability among samples, in this case the biological variation among specimens, on a zero to one scale. Values closer to one indicate higher repeatability, and values closer to zero indicate lower repeatability (Arnqvist & Mårtensson, 1998; Fruciano, 2016).

**Table 1:** Pairwise analysis of landmark datasets comparing Procrustes ANOVA residual *R* ^2^ percentages (ProcANOVA *R* ^2^ [%]) and repeatability among datasets, absolute differences among comparisons in cross‐validated linear discriminant analysis predicted group membership error (PGM Error Change [%]), and differences among comparisons in the percent of predicted group membership changes among individual Project 23 fossils of unknown species affinity (Fossil PGM Change [%])

Dataset comparisons (trials)	Error source quantified	Repeatability	ProcANOVA *R* ^2^ (%)	PGM Error Change (%)	Fossil PGM Change (%)
Dinolite_NoTilt_EO_**T1**–Dinolite_NoTilt_EO_**T2**	Intraobserver	0.80	6.99	3.6	9.7
Dinolite_NoTilt_NO_**T1**–Dinolite_NoTilt_NO_**T2**	Intraobserver	0.61	13.74	3.7	22.6
Nikon_NoTilt_EO_**T1**–Nikon_NoTilt_EO_**T2**	Intraobserver	0.64	12.69	0.0	35.5
Nikon_NoTilt_NO_**T1**–Nikon_NoTilt_NO_**T2**	Intraobserver	0.61	13.70	3.3	22.6
*Mean among all intraobserver dataset comparisons*		*0.66*	*11.78*	*2.7*	*22.6*
Dinolite_NoTilt_**EO**_T1–Dinolite_NoTilt_**NO**_T1	Interobserver	0.21	31.01	20.6	32.3
Dinolite_NoTilt_**EO**_T2–Dinolite_NoTilt_**NO**_T2	Interobserver	0.19	32.24	20.7	16.1
Dinolite_NoTilt_**EO**_T1–Dinolite_NoTilt_**NO**_T2	Interobserver	0.18	32.66	24.3	25.8
Dinolite_NoTilt_**EO**_T2–Dinolite_NoTilt_**NO**_T1	Interobserver	0.21	31.32	19.8	32.3
Nikon_NoTilt_**EO**_T1–Nikon_NoTilt_**NO**_T1	Interobserver	0.58	14.84	7.3	32.3
Nikon_NoTilt_**EO**_T2–Nikon_NoTilt_**NO**_T2	Interobserver	0.25	28.87	10.6	32.3
Nikon_NoTilt_**EO**_T1–Nikon_NoTilt_**NO**_T2	Interobserver	0.42	21.31	10.6	29.0
Nikon_NoTilt_**EO**_T2–Nikon_NoTilt_**NO**_T1	Interobserver	0.39	22.78	7.3	35.5
*Mean among all interobserver dataset comparisons*		*0.30*	*26.88*	*15.2*	*29.5*
**Dinolite**_NoTilt_EO_T1–**Nikon**_NoTilt_EO_T1	Device	0.54	16.56	6.4	35.5
**Dinolite**_NoTilt_EO_T2–**Nikon**_NoTilt_EO_T2	Device	0.65	12.36	2.8	32.3
**Dinolite**_NoTilt_EO_T1–**Nikon**_NoTilt_EO_T2	Device	0.63	12.87	6.4	41.9
**Dinolite**_NoTilt_EO_T2–**Nikon**_NoTilt_EO_T1	Device	0.55	15.97	2.8	25.8
**Dinolite**_NoTilt_NO_T1–**Nikon**_NoTilt_NO_T1	Device	0.30	26.65	6.9	29.0
**Dinolite**_NoTilt_NO_T2–**Nikon**_NoTilt_NO_T2	Device	0.35	24.11	7.3	32.3
**Dinolite**_NoTilt_NO_T1–**Nikon**_NoTilt_NO_T2	Device	0.39	22.72	3.6	29.0
**Dinolite**_NoTilt_NO_T2–**Nikon**_NoTilt_NO_T1	Device	0.27	27.84	10.6	35.5
*Mean among all imaging device dataset comparisons*		*0.46*	*19.89*	*5.9*	*32.7*
Dinolite_**NoTilt**_EO_T1–Dinolite_**Tilted**_EO_T1	Presentation	0.44	20.36	20.6	45.2
Dinolite_**NoTilt**_EO_T2–Dinolite_**Tilted**_EO_T1	Presentation	0.45	20.08	17.0	41.9
*Mean among all presentation dataset comparisons*		*0.45*	*20.22*	*18.8*	*43.6*

Note

Datasets are paired according to the respective error sources they quantify. Analyzed levels (i.e., error sources) of each comparison are bolded, and mean differences among datasets for each level are italics. Dataset name segments indicate the following: Dinolite = images photographed with a Dino‐Lite camera are included; Nikon = images photographed with a Nikon camera are included; NoTilt = specimens photographed from a standardized orientation are included; Tilted = specimens photographed from haphazardly tilted orientations are included; EO = landmark configurations digitized by the experienced observer are included; NO = landmark configurations digitized by the new observer are included; T1 = landmark data from the first digitizing iteration of the respective image set and observer are included; and T2 = landmark data from the second digitizing iteration of the respective image set and observer are included.

### 2.5 Quantifying measurement error impacts on classification statistics

To determine how source‐specific measurement error impacts *Microtus* species classification, we ran LDAs on each of the nine GPA‐transformed landmark trial datasets using the lda function in the R package “MASS” (version 7.3, Venables & Ripley, 2002). Forty‐two *x*, *y* coordinates from the 21 digitized landmarks were used as predictor variables to classify each specimen into a predicted species group. We used leave‐one‐out cross‐validation to determine the percentage of specimens correctly classified within their respective species groups since it reduces standard LDA group overfitting (Kovarovic et al., 2011). Prior probabilities of group membership were assigned using the default lda argument based on the proportion of group samples which, in this case, are nearly equal due to similar sample sizes among species. Linear discriminant analysis predicted group membership (PGM) error percentages were calculated for each landmark dataset by dividing the number of misclassified individuals across all five species by the total number of individuals (*n* = 247) multiplied by 100. Differences in absolute PGM error percentages among the 22 pairwise dataset comparisons were then recorded. Additionally, a stepwise discriminant analysis was performed on a subset of trials to evaluate whether the significance of different landmark variables for discriminating extant *Microtus* species groups changes among data acquisition iterations. A standard LDA was performed in all other cases unless specified otherwise.

Next, a set of 31 fossil *Microtus* m1 images of unknown species identity was digitized by the EO, using the same 21‐landmark protocol, and appended to each dataset of recent *Microtus* specimens to evaluate error impacts on the PGM of unknown specimens. Fossil specimens included mostly isolated m1s and were photographed with the same Dino‐Lite camera as recent *Microtus* specimens. Each of the nine recent *Microtus* landmark datasets served as a unique discriminant function training set to classify the unknown fossils into one or more of the five extant species groups. All fossil specimens are from Project 23, Deposit 1, at Rancho La Brea in Los Angeles, CA and are late Pleistocene in age (~46,000 to ~31,000 radiocarbon years before present; Fox, Takeuchi, Farrell, & Blois, 2019; Fuller et al., 2020). Due to their geographic and temporal location, it is unlikely that the fossils belong to a species of *Microtus* other than the five included in our LDA training sets. Linear discriminant analyses were run on landmark coordinate variables of each dataset with fossils entered as unknowns, and the PGM of each fossil specimen in each trial was recorded.

### 2.6 Species occurrence likelihood

Since LDA of western North American vole species is <100% accurate (McGuire, 2011), it may be difficult to determine whether some PGMs are “real” or altered by error within the LDA training set, especially when the number of individuals assigned to a species group is small. Therefore, we consider a species occurrence “likely” if the percentage of unknown individuals classified to a species group, relative to the total number of individuals within the unknown dataset, exceeds the percentage of cross‐validated classification error within the LDA training set. For example, a dataset that misclassifies 40 of the 247 recent *Microtus* specimens (16.2%) must assign more than 16.2% of the total unknown specimens to a particular species group for that species to be considered “likely present.” Thus, if 15 of the 31 unknown specimens were assigned to *M. californicus* (48.3%), four were assigned to *M. montanus* (12.9%), and two were assigned to *M. townsendii* (6.5%); only *M. californicus* would be considered “likely present” since the percentage of specimens assigned to the other two species falls within the range of classification error for that dataset (see Results). To further vet false occurrences, we removed all fossil individuals with PGM posterior probabilities <.95 prior to these likelihood calculations.

## 3 RESULTS

### 3.1 Source‐specific variation

Pairwise and nested Procrustes ANOVA of the landmark datasets show that all potential error sources generate significant error (Tables 1 and 2). Of the four sources, interobserver error is the most substantial and explains ~27% of the variation among datasets, on average, in pairwise comparisons (see *R* ^2^ values, Table 1). The next greatest sources of error in the pairwise comparisons are seen among specimen presentations and imaging devices, both of which explain ~20% of the variation on average, followed by intraobserver error which explains ~12% of pairwise variation on average (Table 1). The combined model shows similar patterns for different contributions to variation (Table 2): Interobserver error accounts for ~21% of the variation, whereas device and intraobserver error explain ~8% and 10% of among‐dataset variation, respectively, in the nested Procrustes ANOVA model. These data acquisition error sources together explain ~39% of the total variation among datasets, while biological variation among individuals and species explains ~53% and ~8% of the total variation, respectively (Table 2). Error source‐specific repeatability is similar to patterns of Procrustes ANOVA *R* ^2^ variation—interobserver pairwise comparisons are the least repeatable overall (mean = 0.30), followed by presentation comparisons (mean = 0.45), device comparisons (mean = 0.46), and intraobserver comparisons (mean = 0.66; Table 1).

**Table 2:** Nested Procrustes ANOVA summary statistics of variation attributed to biological factors (i.e., among species and individual specimens) and three data acquisition error sources: imaging device, interobserver digitization, and intraobserver digitization across eight landmark datasets

Error source	*df*	SS	*R* ^2^	*Z*	*p* Value
Species	4	0.7619	0.07818	16.265	.001
Individuals	247	5.1919	0.53276	28.423	.001
Device	242	0.7302	0.07493	11.767	.001
Interobservers	494	2.0661	0.21201	24.689	.001
Intraobservers	988	0.9953	0.10213	29.826	.001
Total	1,975	9.7453			

Note

The “Dinolite_Tilted_EO_T1” dataset was not included since it did not fit into the nested analytical hierarchy (Figure A1).

### 3.2 Classification accuracy

Cross‐validated PGM error varies substantially among the 22 pairwise LDA comparisons with 0%–24.3% discrepancies in absolute error among landmark datasets (Table 1). Contrary to Procrustes ANOVA results, differences in PGM error are greatest between datasets of differential presentation (i.e., between tilted vs. nontilted specimen images), which exhibit a mean pairwise PGM accuracy difference of 18.8% (Table 1). Datasets digitized by different observers generate the next greatest amount of variation in pairwise PGM error overall (mean difference = 15.2%) followed by device error and intraobserver error which yield mean PGM accuracy shifts of 5.9% and 2.7%, respectively, among pairwise comparisons (Table 1). Cross‐validated PGM error of all extant *Microtus* species ranges from 13.8% to 38.1% among the nine datasets, with a mean error of 26.3% among datasets overall (Table 3). Predicted group membership errors are substantially lower in non‐cross‐validated analyses of the same datasets (PGM error = 2.8%–20.6%, Table A1), likely due to PGM overfitting (Kovarovic et al., 2011). Variables selected for discriminating extant *Microtus* species groups in stepwise discriminant analysis also vary among datasets, even among datasets collected with the same imaging device and digitized by the same observer (Table A2).

**Table 3:** Cross‐validated linear discriminant analysis (LDA) classification statistics of 31 fossil *Microtus* m1s from Project 23 at Rancho La Brea

Landmark dataset	*Microtus californicus*	*Microtus longicaudus*	*Microtus montanus*	*Microtus oregoni*	*Microtus townsendii*	Error (%)
Dinolite_NoTilt_EO_T1	*20*	1	0	2	1	13.8
Dinolite_NoTilt_EO_T2	*14*	1	0	2	0	17.4
Nikon_NoTilt_EO_T1	6	3	0	1	0	20.2
Nikon_NoTilt_EO_T2	3	*8*	0	1	0	20.2
Nikon_NoTilt_NO_T1	10	1	0	1	0	27.5
Nikon_NoTilt_NO_T2	6	3	0	0	0	30.8
Dinolite_NoTilt_NO_T1	*12*	1	0	0	0	34.4
Dinolite_Tilted_EO_T1	3	1	0	0	0	34.4
Dinolite_NoTilt_NO_T2	*13*	0	0	0	0	38.1
Mean	9.7	2.1	0	0.8	0.1	26.3
Range	3–20	0–8	0	0–2	0–1	13.8–38.1

Note

Column values indicate the number of fossil specimens assigned to each extant species per landmark dataset. Specimens with predicted group membership probabilities <.95 are not included. “Error (%)” indicates the percentage of recent *Microtus* specimens (*n* = 247) misclassified within the LDA training set. Italicised values mark a species' presence as “likely” according to the accuracy of its respective LDA training set. See main text and Table 1 for explanations of species likelihood calculations and dataset naming, respectively.

### 3.3 Predicted group membership replicability

Predicted group memberships of unknown *Microtus* fossils from Project 23, Deposit 1, at Rancho La Brea vary substantially among the nine landmark datasets (Tables 1 and 3; Table A1). Individual fossil PGM discrepancies range from 9.7% to 45.2% among the 22 pairwise dataset comparisons (Table 1). As with differences in PGM error of recent specimens, PGM differences among the unknown fossils is greatest between trials of differential presentation (mean PGM variation = 43.6%) followed by different imaging devices (32.7%), different observers (29.5%), and within observers (22.6%; Table 1). *Microtus californicus* is the most frequently assigned species; the number of fossil individuals classified as *M. californicus* with predicted probabilities >.95 ranges from three to 20 among datasets (Table 3). *Microtus californicus* is considered “likely present” in four of the nine datasets according to our likelihood criterion. *Microtus longicaudus* is the second most frequently assigned species; 0 to eight individuals are classified as this species within datasets after probability vetting, and it is considered “likely present” in one of the nine datasets (Table 3). Individual specimens assigned to the other three species range from 0, 0–2, and 0–1 for *M. montanus*, *M. oregoni*, and *M. townsendii*, respectively, after probability vetting. None of those species are considered “likely present” in any dataset using our likelihood criterion (Table 3). Relative proportions of fossil individuals assigned to each species group are similar across datasets when posterior probability vetting and leave‐one‐out cross‐validation is not employed, though the number of individuals retained in each species group is greater. The number of datasets with *M. californicus* and *M. longicaudus* considered likely present increases to nine and eight, respectively, using this procedure (Table A1).

## 4 DISCUSSION

### 4.1 Landmark data acquisition error

We have shown that error introduced from various landmark data acquisition sources can be substantial and, in some cases, explains >30% of nonbiological variation among datasets (Tables 1 and 2). This is concerning for geometric morphometric analyses aiming to quantify shape change among biological groups—including studies of taxonomy, functional ecology, and population history—because large amounts of error may impact hypothesis testing outcomes and/or lead to erroneous interpretations of focal‐group relationships (Buser et al., 2018). It is therefore necessary to identify source‐specific causes of error and establish protocols to mitigate error as much as possible.

**Figure 2:**
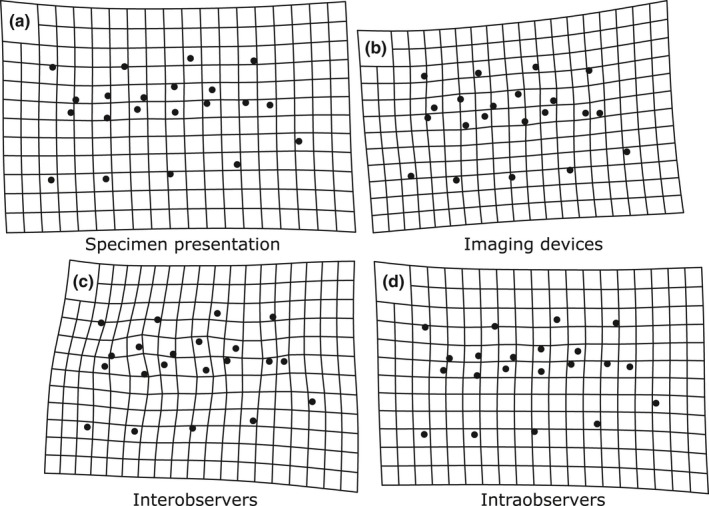
Thin‐plate spline deformation grids illustrating mean shape changes between reference dataset and target dataset landmark configurations of *Microtus* lower first molars. (a) Specimen presentation impacts on overall landmark coordinate shape between datasets “Dinolite_Notilt_EO_T1” and “Dinolite_Tilt_EO_T1.” (b) Imaging device impacts on overall landmark coordinate shape between datasets “Nikon_NoTilt_NO_T2” and “Dinolite_NoTilt_NO_T2.” (c) Interobserver impacts on overall landmark coordinate shape between datasets “Dinolite_NoTilt_EO_T2” and “Dinolite_NoTilt_NO_T2.” (d) Intraobserver impacts on overall landmark coordinate shape between datasets “Dinolite_NoTilt_EO_T1” and “Dinolite_NoTilt_EO_T2.” See Table 1 for dataset name explanations

We find interobserver error to be greatest among datasets, overall, followed by error among specimen presentations, within observers, and among different types of imaging equipment (Tables 1 and 2; Figure 2), though the relative importance of device versus intraobserver error differs among pairwise versus nested Procrustes ANOVA analyses (Tables 1 and 2). Such discrepancies are not unexpected since intraobserver error is an inextricable component of device error, presentation error, and interobserver error in pairwise analysis, and therefore, its impact is best captured by the nested analysis. In all cases, variation attributed to data acquisition error sources is less than biological variation among individuals but greater than or approximately equal to variation among species (Tables 1 and 2). These findings generally agree with quantifications of interobserver and device error in other studies (e.g., Fruciano et al., 2017; Robinson & Terhune, 2017). For example, Fruciano et al. (2017) found interobserver error to be greater than device error or biological asymmetry, explaining up to 10.2% and 5.4% of the variation within datasets, respectively. Similarly, Robinson and Terhune (2017) found observer error to be the greatest nonbiological source of variation. Interobserver variation in our study may have been exacerbated by differences in digitizing experience among operators, and device error may be elevated by residual presentation error despite controlling for this. Indeed, pairwise Procrustes ANOVA of datasets digitized by the EO and NO yielded considerable differences in *R* ^2^ and repeatability, with less error and higher repeatability of datasets digitized by the EO, suggesting that experience does reduce digitizing error here. Mean intraobserver *R* ^2^ is 9.84% and 13.72%, and repeatability is 0.72 and 0.61, for the EO and NO, respectively (Table 1). Changes in specimen presentation yield the second greatest amount of landmark data variation in pairwise comparisons (Table 1). Although presentation error may have been exacerbated by the intentional titling of specimens in our treatment, Fruciano (2016) also found presentation‐based variation to be significant and substantially greater than intraobserver error on 2D landmark configurations of fish body shape.

### 4.2 Impacts on group classification statistics

**Figure 3:**
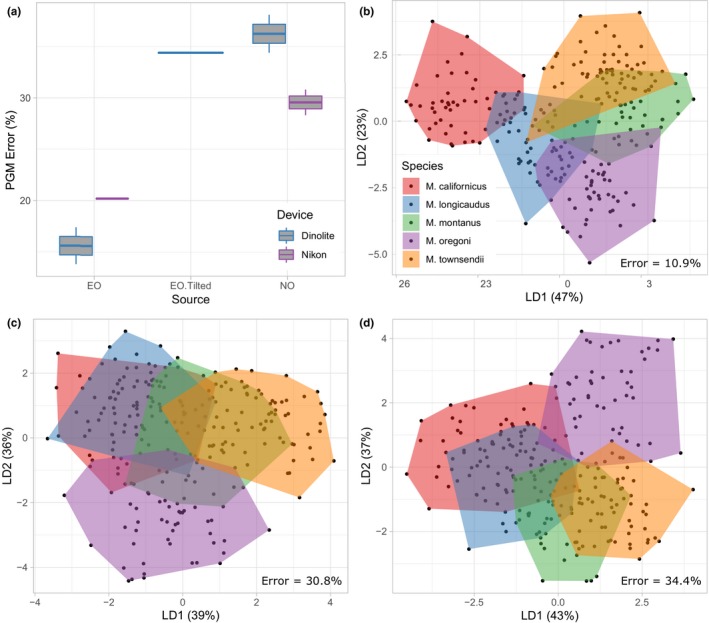
(a) Boxplot of linear discriminant analysis predicted group membership error percentages using leave‐one‐out cross‐validation across all extant *Microtus* species for each dataset in this study (*n* = 9) grouped by observer, imaging device, and specimen orientation. See Table 3 for error values generated from individual datasets. (b–d) Plot of linear discriminant functions one (LD1) and two (LD2) from a subset of the nine landmark datasets: (b) EO intraobserver mean of coordinates “Dinolite_NoTilt_EO_T1” and “Dinolite_NoTilt_EO_T2,” (c) NO intraobserver mean of coordinates “Dinolite_NoTilt_NO_T1” and “Dinolite_NoTilt_NO_T2,”and (d) “Tilted” specimen presentations “Dinolite_Tilt_EO_T1.” All 247 recent *Microtus* individuals are grouped according to species affinity: “Mc” = *Microtus californicus*, “Ml” = *Microtus longicaudus*, “Mm” = *Microtus montanus*, “Mo” = *Microtus oregoni*, and “Mt” = *Microtus townsendii*. “Error” = the percentage of cross‐validated predicted group membership error across all species

As with Procrustes ANOVA variation, LDA PGM error of extant *Microtus* varies substantially among datasets, with up to 24% variation in absolute PGM accuracy among pairwise trial comparisons (Table 1). Unlike Procrustes ANOVA results, however, PGM error changes were greatest among specimens of differential presentation (mean PGM error difference = 18.8% among tilted and nontilted trials) rather than among observers (mean PGM error difference = 15.2% between EO and NO trials; Table 1). Procrustes ANOVA variation and LDA error variation are both used as proxies of error in this study; however, they are not necessarily equivalent. Procrustes ANOVA variation reflects changes in landmark precision among datasets and PGM error variation reflects changes in landmark accuracy relative to the biological loci and groups of interest, which may partly explain these discrepancies. As with Procrustes ANOVA *R* ^2^, PGM error and pairwise differences in absolute PGM error were lower among EO datasets and greater among NO datasets overall (Tables 1 and 3; Figure 3a). Mean cross‐validated PGM error variation of pairwise intraobserver comparisons, excluding tilted trials, is 1.8% and 3.5% among EO and NO trials, respectively (Table 1), and the mean PGM error among eight datasets digitized by each author, excluding the tilted trial, is 17.9% and 32.7% for the EO and NO, respectively (Table 3, Figure 3a). These results suggest that, in this case, digitizing experience improves downstream classification accuracy in addition to increasing landmark precision. In future studies, it would be informative to test the rate at which landmark accuracy and precision improve with experience by conducting further EO and NO digitizing iterations.

The greatest difference in PGM of fossil specimens is observed among pairwise comparisons of different presentations followed by different imaging devices, observers, and iterations within observers (Table 1). Unlike recent specimens of known species affinity, experience‐based intraobserver variation in fossil PGM is similar among observers and trials overall (mean fossil PGM change = 22.6% among both EO and NO trials, Table 1). Pairwise differences in fossil PGM are often large, ranging from 9.7% to 45.2%, even when Procrustes ANOVA *R* ^2^ values and/or extant species PGM differences among pairwise comparisons are small (e.g., between Nikon_NoTilt_EO_T1 and Nikon_NoTilt_EO_T2, Table 1). Data of unknown specimens may be especially sensitive to measurement error because they are often acquired, appended, and/or analyzed only after a meaningful group‐binning protocol has been established among training groups (e.g., Cassini, 2013; De Meulemeester et al., 2012; Figueirido, Martín‐Serra, Tseng, & Janis, 2015; this study). Thus, data acquisition processes and their associated errors may be repeated during unknown specimen data collection, which may exacerbate the amount of artificial variation present among unknowns relative to specimens in the training set. For example, replicating the orientation of recent specimen teeth projected from within jaws could be difficult when projecting isolated teeth of fossil specimens. Indeed, our data show that orientation changes among specimens captured in 2D images can profoundly impact recent and fossil specimen classification statistics (Table 1; Figure 3b,d). Differences in digitizing personnel and/or imaging instruments used to obtain recent and fossil specimen data could accentuate those errors.

Data acquisition error is not only problematic for evaluating the number of specimens classified within a group, but it can also lead to erroneous inferences of taxonomic occurrences at sites when PGM is performed on specimens of unknown taxonomic affinities. Such issues are most likely to arise when comparing morphologically similar groups and/or when PGM error of the training set is large. For example, it may be difficult to determine whether the fossil *M. longicaudus*, *M. montanus*, *M. oregoni*, and *M. townsendii* predicted as present by some LDA training sets in our study actually occur within Project 23, Deposit 1, at Rancho La Brea or whether the few individuals assigned to those species groups are simply an artifact of PGM error (Table A1). Increasing sample sizes of training and unknown specimen groups, rerunning analyses on multiple landmark iterations, and employing error‐based occurrence and PGM probability vetting can help elucidate which group occurrences are likely real and which are likely attributable to nonbiological error sources (e.g., Table 3). Even with those measures, however, the presence of some groups may be uncertain depending on analysis‐specific intergroup similarity and PGM accuracy. For example, the few individuals assigned to *M. montanus*, *M. oregoni*, and *M. townsendii* must be viewed with skepticism since they fall within the range of cross‐validated PGM error of all recent specimen training sets using our species likelihood criterion (Table 3). However, *M. longicaudus* is considered likely present in one or eight of the nine landmark datasets depending on which likelihood criterion is used (Table 3, Table A1). This is interesting since an isolated, high‐elevation population of *M. longicaudus* is present in the San Bernardino mountains today (Patterson et al., 2003). Nevertheless, the occurrence of *M. longicaudus* in Deposit 1 at Rancho La Brea is uncertain until a larger fossil sample size is acquired.

### 4.3 Relationships among error proxies and data replicability

Our results indicate that some landmark data acquisition sources contribute relatively large amounts of variation across error proxies (e.g., interobserver error quantified by Procrustes ANOVA and LDA; Tables 1 and 2; Figure 2). However, all error sources are significant and impact classification statistics of recent and fossil *Microtus* specimens to some extent (Table 1; Figure 3). The fact that source‐specific measurement error is significant, alone, does not indicate that it will substantially impact downstream classification results. For example, Fruciano et al. (2020) found significant differences in fish body shape attributed to different preservation treatments. However, the impact of preservation treatment on LDA fish‐group classification was minimal. The authors attributed that discrepancy to differences in shape change direction between the fish groups of interest and preservation‐based error (i.e., shape change due to preservation and biological shape change was not parallel in that system; Fruciano et al., 2020).

**Figure 4:**
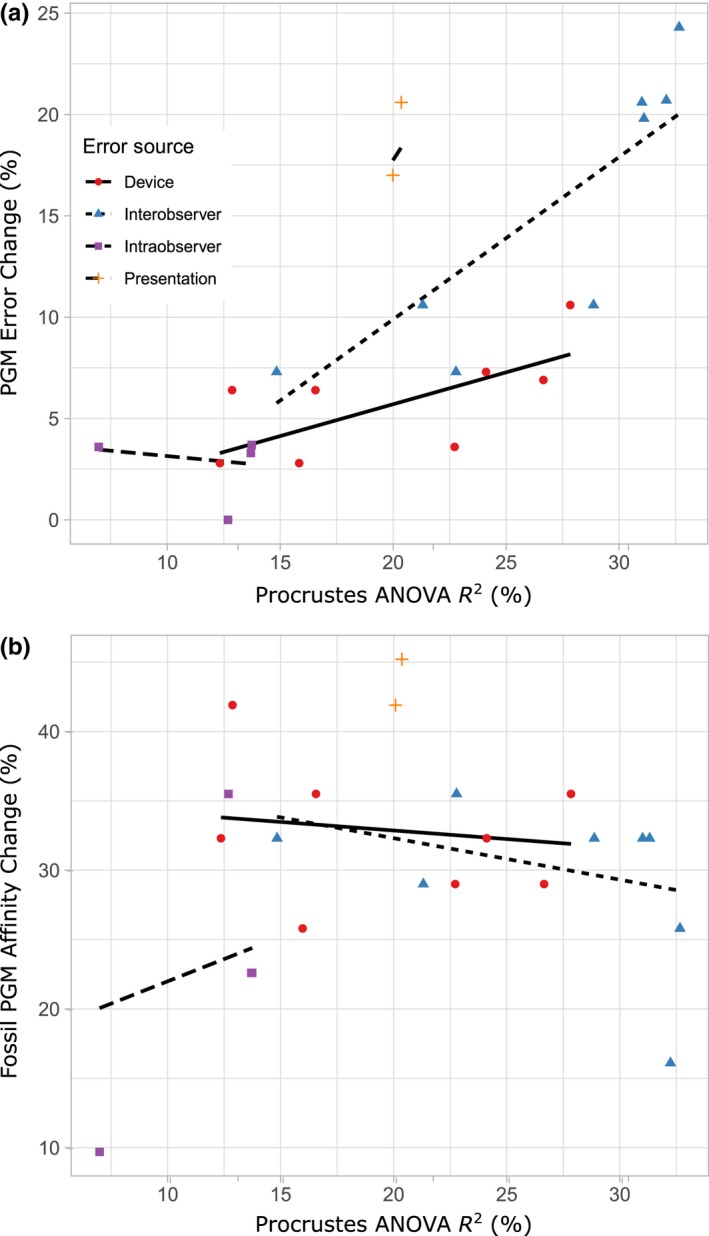
(a) Linear mixed model of pairwise Procrustes ANOVA residual *R* ^2^ (error) percentages and pairwise differences in linear discriminant analysis (LDA) predicted group membership (PGM) absolute error percentages of extant *Microtus* species. (b) Plot of Procrustes ANOVA residual *R* ^2^ percentages and percentages of LDA PGM affinity differences of fossil *Microtus* from Rancho La Brea, Project 23, Deposit 1 using the same pairwise dataset comparisons as (a). Points are colored according to error source, and all pairwise dataset comparisons are listed in Table 1. Models were run using the lmer function in the R package “lme4” (Bates, Mächler, Bolker, & Walker, 2015)

In our study, extant *Microtus* PGM accuracy and consistency generally align with Procrustes ANOVA variation such that pairwise comparisons of datasets with lower *R* ^2^ values and higher repeatability values exhibit greater PGM agreement (Table 1, Figure 4a). This trend suggests that measurement error in our system alters shape in a similar direction to biological shape variation among *Microtus* species. However, the trend does not hold for pairwise comparisons of fossils. Predicted group membership disagreement is substantial for most pairwise comparisons; though, no obvious relationship is observed between Procrustes ANOVA R^2^/repeatability values and fossil PGM affinity differences (Table 1, Figure 4b). The latter trend is possibly due to the additional data acquisition phases, and thus greater error potential, inherent of classifying unknowns as mentioned. One notable exception to the overall trend of recent and fossil specimen pairwise data is observed in presentation‐based error. Procrustes ANOVA variation and repeatability among tilted versus nontilted trials are moderate relative to the other three error sources (*R* ^2^ = 20.1%–20.4%, repeatability = 0.44–0.45; Table 1; Figure 2b). Major discrepancies occur, however, in PGM accuracy of recent specimens and PGM affinity of fossil specimens between tilted and nontilted datasets (Table 1; Figures 3b,d and 4). Conversely, pairwise intraobserver PGM differences, and to a lesser extent pairwise imaging device PGM differences, are much lower than pairwise presentation and interobserver PGM differences relative to their Procrustes ANOVA *R* ^2^ and repeatability values overall (Table 1, Figure 4a). In other words, measurement error attributed to different devices and within observers does not have as strong of an effect on classification results as measurement error between specimen presentations and among observers. This suggests that the direction of biological shape variation among *Microtus* species is more dissimilar to the direction of artificial shape variation attributed to device and intraobserver differences than it is to presentation and interobserver differences.

While reduced pairwise PGM error discrepancies among devices and within observes may be caused by differences in biological and artificial shape change directionality, elevated pairwise PGM error among different specimen presentations relative to other error sources could be explained, in part, by the different proxies used to quantify error since pairwise Procrustes ANOVA comparisons quantify landmark precision, and LDA PGMs quantify landmark accuracy. However, the inconsistency of presentation error quantified among those proxies is far greater than that of any other error source (Table 1, Figure 4) so it is unlikely that this is entirely explained by the different proxies of error. Another possible explanation for the presentation error discrepancy observed in our study is image distortion‐facilitated changes in specimen landmark configurations. Rotational changes among 3D specimens in “tilted” trials may distort certain tooth loci captured in 2D images. Such distortions would then displace subsequent landmarks on those loci. Although orientation changes among landmark configurations are mitigated during GPA, the generalized least‐squares algorithm that aligns the configurations to a common coordinate system does not adjust error based on individual point variation. Rather, corrections are distributed randomly across the entire configuration to reduce residual variation of less precise landmarks and increase variation of more precise landmarks to minimize error overall (von Cramon‐Taubadel, Frazier, & Lahr, 2007). This “spreading” of landmark coordinate error during GPA, termed the “Pinocchio effect” (Chapman, 1990; von Cramon‐Taubadel et al., 2007), may alter total specimen shape and thus biological variation among specimens captured via landmarks. The Pinocchio effect could be particularly detrimental for statistical grouping analyses because some shape variables are more relevant for group separation than others. For example, variables that are highly inconsistent within groups are not likely to be selected for LDA since variables that maximize among‐group separation are preferentially selected (Kovarovic et al., 2011). Distributing the error of highly variable landmarks (e.g., those facilitated by presentation inconstancies) across all landmarks may reduce overall error quantified by analyses of variance, but also inhibit the discriminatory power of classification analyses since the most significant among group‐separating variables may be altered by doing so. Indeed, stepwise LDA indicates that variables selected for discriminating *Microtus* species are dissimilar among tilted versus nontilted trials (Table A2). It is perhaps relevant that differences between variables entered in stepwise LDA of tilted versus nontilted trials often occur among landmarks positioned on tooth extremities (e.g., Landmarks 3, 13, and 21; Figure 1; Table A2), which are closest to the image edges where distortion is generally greatest (Zelditch et al., 2004).

Overall, these results indicate that GM classification results of morphologically similar taxa are not always replicable due, in part, to multiple sources of data acquisition error. No two iterations among the nine resampled specimen datasets of this study exhibit the same intragroup classification results, and many datasets yield dissimilar predictions of fossil species occurrences (Table 3, Table A1). However, our findings may be different from those of other studies since the impact of measurement error on data replicability will likely vary based on analysis‐specific objectives, inter‐ and intragroup similarity, and statistical classification accuracy (Robinson & Terhune, 2017). For example, small to moderate amounts of measurement error may be negligible for studies classifying organisms at the family level because among‐group biological variation may surpass any artificial variation introduced to that system. Similarly, small amounts of measurement error and classification inaccuracy may be acceptable for quantifying interspecific occurrences, but not for quantifying intraspecific abundances within the same system. The amount of introduced error that surpasses an acceptable threshold will likely vary case‐by‐case depending on the respective analytical design, focal system, and questions/objectives of the study (Fruciano, 2016; Robinson & Terhune, 2017). Nevertheless, there are general measures that can be taken to mitigate error in any system.

### 4.4 Mitigating error and error impacts

It is impossible to eliminate GM error completely (Fruciano, 2016), but there are several ways to lessen the amount of error introduced. Presentation error, for example, has the most egregious impact on group classification replicability in our study. Although this error may have been exacerbated by intentional tilting of specimens in the “tilted” data acquisition trial, our findings indicate that presentation error can impact landmark‐based classification statistics considerably if not properly managed (Table 1; Figures 3 and 4). Of further concern is the fact that this error source is less detectable through common error‐quantifying methods (i.e., Procrustes ANOVA) than other data acquisition sources that introduce large amounts of error (e.g., interobservers) (Table 1; Figure 2), possibly due to the Pinocchio effect of GPA. Presentation error can be mitigated by using 3D GM which bypasses error associated with dimensional loss (Buser et al., 2018; Cardini, 2014). Three‐dimensional GM technology has improved greatly over the past two decades with respect to its data resolution and cost (Cardini, 2014). High‐resolution 3D analyses that were previously restricted to larger specimens are becoming increasingly applicable to small objects (e.g., Cornette, Baylac, Souter, & Herrel, 2013), such as the *Microtus* molars evaluated in our study. However, 2D GM will be more feasible for some projects since it is generally more affordable and can be conducted faster and with more versatile analytical equipment than 3D GM (Cardini, 2014). Researchers interested in conducting 2D GM analyses should therefore standardize specimen projection orientations as much as possible to mitigate presentation error.

For 2D and 3D GM analyses, we recommend that researchers avoid mixing observers due to the considerable amount of digitization error that can be generated among them (Tables 1, 2; Figures 2 and 4). After such precautions have been taken, determining the fidelity of statistical results, and/or whether the amount of error introduced is negligible, will be study‐specific and dependent on intergroup data similarity and the overall accuracy of the analysis. Our findings suggest that, in general, groups with low numbers of unknown individuals assigned to them should be considered with caution, especially when classification accuracy and/or among‐group variation is relatively low (Tables 2, 3, Figure 3, Table A1). Including relatively large sample sizes, posterior probability thresholds, and multiple (intraobserver) digitizing iterations may help infer group occurrence fidelity.

In conclusion, GM measurement error from different landmark data acquisition sources has the potential to obscure biologically meaningful shape variation, facilitate statistical misclassification, and negatively impact data replicability. We do not discourage using GM for biological group classification since it is among the most powerful techniques available for quantifying shape and shape variability among groups. Rather, we hope this study provides an informative, if cautionary, example of why GM error should be mitigated to the greatest feasible extent. After precautions have been taken to reduce measurement error, repeated measurements and statistical evaluations can be employed to facilitate decisions of whether the amount of residual error is acceptable for study‐specific research objectives.

## CONFLICT OF INTEREST

None declared.

## AUTHOR CONTRIBUTIONS

N. Fox designed the study, led the acquisition, analysis, and interpretation of data, and drafted the article. J. Veneracion helped acquire data and performed data analyses. J. Blois helped design the study, provided analytical support, and revised the manuscript. All authors approved the final version of this manuscript for publication.

## Data Availability

MVZ specimen metadata: https://arctos.database.museum/SpecimenSearch.cfm. Landmark data input files, specimen images, and analytical code are available from the Dryad Digital Repository: https://doi.org/10.6071/M3KD40.

## APPENDIX 1

**Table A1:** Linear discriminant analysis (LDA) classification statistics of 31 fossil *Microtus* m1s from Project 23 at Rancho La Brea using the same landmark datasets reported in Table 3 without leave‐one‐out cross‐valuation or posterior probability vetting

Landmark dataset	*Microtus californicus*	*Microtus longicaudus*	*Microtus montanus*	*Microtus oregoni*	*Microtus townsendii*	Error (%)
Dinolite_NoTilt_EO_T1	*25*	*3*	0	*2*	*1*	2.8
Dinolite_NoTilt_EO_T2	*24*	*5*	0	*2*	1	5.7
Nikon_NoTilt_EO_T1	*16*	*9*	*3*	*3*	0	9.3
Nikon_NoTilt_EO_T2	*16*	*14*	0	1	0	9.3
Nikon_NoTilt_NO_T1	*22*	*5*	0	3	1	12.2
Nikon_NoTilt_NO_T2	*19*	*9*	0	2	1	15.0
Dinolite_NoTilt_NO_T1	*20*	*7*	0	4	0	18.6
Dinolite_Tilted_EO_T1	*17*	*11*	1	2	0	14.6
Dinolite_NoTilt_NO_T2	*25*	5	0	1	0	20.6
Mean	20.4	7.6	0.4	2.2	0.3	12.0
Range	16–25	3–14	0–3	1–4	0–1	2.8–20.6

Note

Column values indicate the number of fossil specimens assigned to each extant species per landmark dataset. Italicised values mark a species' presence as “likely” according to the accuracy of its respective LDA training set. See main text and Table 1 for explanations of species likelihood calculations and dataset naming, respectively.

**Table A2:** Comparison of landmarks entered in stepwise discriminant analysis of the five extant *Microtus* species between digitizing iterations of the experienced observer using standardized Dino‐Lite presentation images (Dinolite_NoTilt_EO_T1 and Dinolite_NoTilt_EO_T2) and haphazardly tilted Dino‐Lite presentation images (Dinolite_Tilted_EO_T1)

Landmark	Dinolite_NoTilt_EO_T1	Dinolite_NoTilt_EO_T2	Dinolite_Tilted_EO_T1
LM1	Included	—	—
LM2	Included	Included	Included
LM3	—	—	Included
LM4	—	Included	Included
LM5	Included	—	—
LM6	—	Included	Included
LM7	Included	Included	Included
LM8	Included	Included	Included
LM9	—	Included	Included
LM10	—	Included	Included
LM11	Included	—	Included
LM12	Included	—	—
LM13	Included	Included	—
LM14	Included	Included	—
LM15	—	Included	—
LM16	—	—	Included
LM17	Included	Included	Included
LM18	Included	Included	—
LM19	Included	Included	Included
LM20	Included	Included	Included
LM21	Included	Included	—

Note

Landmarks are labeled as “included” if their *x* coordinate variable, *y* coordinate variable, or both were entered in the analysis. See Table 1 for dataset name explanations.
